# cGAS-STING, inflammasomes and pyroptosis: an overview of crosstalk mechanism of activation and regulation

**DOI:** 10.1186/s12964-023-01466-w

**Published:** 2024-01-09

**Authors:** Jingwen Liu, Jing Zhou, Yuling Luan, Xiaoying Li, Xiangrui Meng, Wenhao Liao, Jianyuan Tang, Zheilei Wang

**Affiliations:** 1 Hospital of Chengdu University of Traditional Chinese Medicine, Chengdu, 610075 China; 2 TCM Regulating Metabolic Diseases Key Laboratory of Sichuan Province, Hospital of Chengdu University of Traditional Chinese Medicine, Chengdu, 610075 China; 3 The Second Hospital of Ningbo, Ningbo, 315099 China; 4 Putuo Hospital, Shanghai University of Traditional Chinese Medicine, Shanghai, 201203 China; 5 Yueyang Hospital of Integrated Traditional Chinese and Western Medicine, Shanghai University of Traditional Chinese Medicine, Shanghai, 200080 China

**Keywords:** cGAS-STING, Inflammasome, Pyroptosis, Inflammation, Crosstalk network, Diseases

## Abstract

**Background:**

Intracellular DNA-sensing pathway cGAS-STING, inflammasomes and pyroptosis act as critical natural immune signaling axes for microbial infection, chronic inflammation, cancer progression and organ degeneration, but the mechanism and regulation of the crosstalk network remain unclear.

**Main body of the abstract:**

Cellular stress disrupts mitochondrial homeostasis, facilitates the opening of mitochondrial permeability transition pore and the leakage of mitochondrial DNA to cell membrane, triggers inflammatory responses by activating cGAS-STING signaling, and subsequently induces inflammasomes activation and the onset of pyroptosis. Meanwhile, the inflammasome-associated protein caspase-1, Gasdermin D, the CARD domain of ASC and the potassium channel are involved in regulating cGAS-STING pathway. Importantly, this crosstalk network has a cascade amplification effect that exacerbates the immuno-inflammatory response, worsening the pathological process of inflammatory and autoimmune diseases. Given the importance of this crosstalk network of cGAS-STING, inflammasomes and pyroptosis in the regulation of innate immunity, it is emerging as a new avenue to explore the mechanisms of multiple disease pathogenesis. Therefore, efforts to define strategies to selectively modulate cGAS-STING, inflammasomes and pyroptosis in different disease settings have been or are ongoing. In this review, we will describe how this mechanistic understanding is driving possible therapeutics targeting this crosstalk network, focusing on the interacting or regulatory proteins, pathways, and a regulatory mitochondrial hub between cGAS-STING, inflammasomes, and pyroptosis.

**Short conclusion:**

This review aims to provide insight into the critical roles and regulatory mechanisms of the crosstalk network of cGAS-STING, inflammasomes and pyroptosis, and to highlight some promising directions for future research and intervention.

## Background

Stimulator of interferon genes (STING) is a cell membrane DNA sensor widely distributed in the endoplasmic reticulum (ER) of mammalian immune cells, which is a vital mediator to regulate innate immune responses. Activation of STING confers host immunity and is key to the clearance of a variety of pathogens, including viruses and bacteria [1–3]. As a DNA recognition receptor, cyclic GMP-AMP synthase (cGAS) recognizes and binds double-stranded DNA (dsDNA) of both foreign and self-origin without sequence differences. cGAS enzymatically converts adenosine triphosphate (ATP) and guanosine triphosphate (GTP) to 2’-3’ cyclic GMP-AMP (cGAMP). cGAMP acts as a second messenger to potently agonize the ER membrane protein STING. STING subsequently recruits and activates TANK-binding kinase 1 (TBK1) to initiate downstream signaling, which in turn promotes the phosphorylation of interferon (IFN) regulatory factor 3 (IRF3), while STING promotes nuclear factor-kappa B (NF-κB) phosphorylation by activating IκB kinase (IKK). IRF3 is then dimerized and translocated to the nucleus with NF-κB to induce *type I* IFN and other cytokines [4–7]. We note that major drug discovery efforts are currently underway to explore and identify agonists of the cGAS-STING pathway as vaccine adjuvants or as anticancer immunostimulants [8–11]. In immunocompetent mice with established syngeneic colon tumors, intravenous administration of a synthetic, non-nucleotide-based diABZI STING agonist exhibits potent anti-tumor activity [12]. Vaccines adjuvated with STING agonists have been shown to elicit potent immune responses against infection and cancer [13]. The natural STING agonist, cGAMP, is a potent adjuvant that improves the immunogenicity of nanoparticulate Influenza A vaccines by enhancing humoral, cellular and mucosal immune responses in mice [14, 15]. In addition, excessive STING activation has been identified as contributing to the progression of various inflammatory diseases [16–19].

Inflammasomes, such as NACHT, LRR, and PYD domains-containing protein 3 (NLRP3) and absent in melanoma 2 (AIM2), initiate the release of pro-inflammatory cytokines upon receipt of danger signals to activate the innate immune response and are essential for the clearance of pathogens or damaged cells. NLRP3 is an intracellular sensor that recognizes a wide variety of microbial motifs, endogenous danger signals and environmental irritants, triggering the formation and activation of the NLRP3 inflammasome. A two-step process of priming and activation is required for NLRP3 inflammasome [20]. In the priming stage, NF-κB is first activated by recognition receptors such as Toll-like receptors (TLRs) that recognize pathogen-associated molecular patterns (PAMPs) or danger signaling molecular patterns (DAMPs), followed by upregulation of NLRP3 and pro-IL-1β [21]. During the activation stage, the inflammasome complex (NLRP3-ASC-caspase-1) is assembled and activated by various inducers, such as viral, bacterial, various interventions [22–24]. Once activated, caspase-1 subsequently functions to result in pyroptosis and cleavage of the proinflammatory cytokines pro-IL-1β and pro-IL-18 into their bioactive forms IL-1β and IL-18, to amplify the inflammatory response [25]. Upon the activation of AIM2 inflammasome, the effector protein caspase-1 is recruited to the complex and cleaves gasdermin D (GSDMD) to release the GSDMD-N fragment, inducing pyroptosis and the release of cellular contents [26]. Pyroptosis is a type of pro-inflammatory programmed cell death that is marked by cell dilation, formation of plasma membrane pores, rapid cell degradation, and the release of inflammatory cytokines [27]. Pyroptosis contributes to the protection of the body from infections such as bacteria, but excessive pyroptosis can lead to chronic inflammation and immune disorders [28–31].

The last decade has witnessed a dramatic appreciation of inflammasomes, pyroptosis and cGAS-STING as critical innate immune components that orchestrate host immune homeostasis. Although inflammasomes, pyroptosis and cGAS-STING are relatively independent innate immune signaling pathways, there is an intracellular signaling network between cGAS-STING, inflammasomes and pyroptosis. In this review, we focus on recent findings regarding the impact of this crosstalk network as a primary driver of inflammatory diseases. We briefly highlight the current state of understanding of signaling through the cGAS-STING, inflammasomes, and pyroptosis pathways, summarize the molecular mechanisms in different pathophysiological contexts, and analyze their involvement in preclinical disease models. On this basis, the key molecular events underlying the crosstalk between cGAS-STING, inflammasomes and pyroptosis were elucidated. In addition, in view of the important role of this crosstalk network in the innate immune response, we also concentrate on the emergence of pharmacological approaches that target the crosstalk network and demonstrate their potential for clinical application. A better understanding of the crosstalk network of cGAS-STING, inflammasomes and pyroptosis will guide the development of therapeutic strategies to combat infectious and inflammatory diseases.

## Inflammasomes and pyroptosis regulate cGAS-STING

### AIM2 inflammasome regulates cGAS-STING

**Fig. 1:**
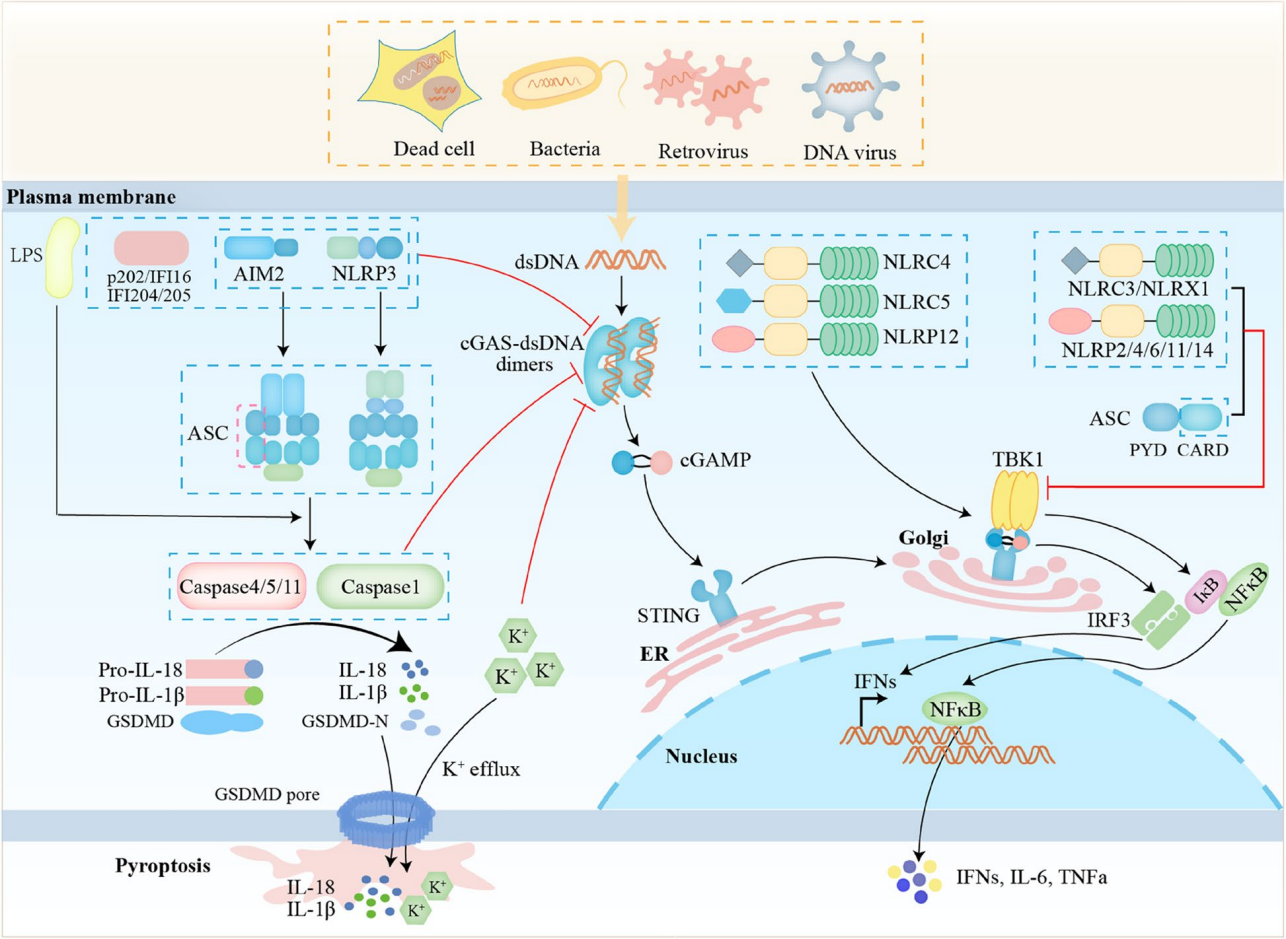
Inflammasomes and pyroptosis regulate cGAS-STING. AIM2 and NLRP3 proteins, AIM2-like receptors, caspase-1, GSDMD, the CARD domain of ASC, potassium channel, and Nod-like receptors are involved in regulating cGAS-STING pathway

The STING-*type I* IFN and AIM2 inflammasome activated by DNA ligands may be crucial to elucidate (Fig. 1). In dendritic cells (DCs) and macrophages deficient in AIM2, ASC, or caspase-1, cGAMP production, STING aggregation, and TBK1 and IRF3 phosphorylation were significantly enhanced upon cytosolic DNA exposure [32], demonstrating that the inhibition of the STING pathway by the AIM2 impacts upstream STING, thus reducing the entire STING pathway activation cascade. Similarly, AIM2 deficiency led to large aggregates of macrophages (CXCR3^+^CD206^+^) activate the STING-TBK1-IRF3/NF-κB pathway in response to dsDNA, resulting in pro-inflammatory cytokines maturation and secretion, including C-X-C motif chemokine 10 (CXCL10), TNFα, and IFN-β [33]. *Mycobacterial* infection of Aim2^−/−^ mice induced the production of large amounts of IFN-β and depressed IFN-γ secretion through suppressing the interaction between STING and downstream TBK1 in macrophages and DCs [34], resulting in higher infection loads and more severe pathology. Thus, these findings suggest that the AIM2 negatively regulates the cGAS-STING-driven production of *type I* IFN upon stimulation with various DNA forms.

### AIM2-like receptors (ALRs) regulate cGAS-STING

ALRs are essential for the *type I* IFN response to endogenous host DNA and determine the course of infections, inflammatory diseases, aging, and cancer [35–37]. Studies have shown that activation of ALRs is associated with host protection following recognition of bacterial DNA, a process that can occur via direct DNA sensing or indirect sensing of pathogen-associated intracellular alterations [36]. Some members of the ALRs gene family were involved in the cGAS-STING pathway (Fig. 1), e.g., IFI204, IFI205 and the human homologue of IFI204, IFI16 [38–40]. Most known ALRs are excellent candidates for innate immune DNA receptors because they have both a pyrin domain, which mediates protein-protein interactions, and a HIN domain, which directly bind to DNA [41]. The pyrin-only protein PYR-A and the HIN-only protein IFI202b are exceptions among the murine ALRs that potently activate STING [38]. The p202 protein encoded by the IFI202 gene, IFI204 and IFI205 have been shown to be negative regulators of AIM2 inflammasome, which cooperated to sense cytosolic dsDNA to produce a strong *type I* IFN response through activation of cGAS-STING [42–44]. IFI202b expression levels likely contribute to mouse strain-specific susceptibility to Theiler’s murine encephalomyelitis virus (TMEV)-induced central nervous system (CNS) lesions [45]. In accordance with this, TMEV-infected IFN-β^−/−^ C57BL/6 mice show an impaired virus elimination capacity and 70% of these mice develop mild demyelination [45]. The mouse ALR IFI205 senses self-DNA derived from retrotransposons in the cytoplasm of macrophages and activates the *type I* IFN signaling pathway via STING [42]. Notably, the p200 family proteins, represented by IFI204, which is well known as an ALR and murine ortholog of IFI16 [46, 47], were markedly induced in bone marrow-derived dendritic cells (BMDCs) after infection by mouse hepatitis coronavirus (MHV), which belongs to the same genus betacoronavirus as SARS-CoV and MERS-CoV to mimic the acute RNA virus infection [48]. Moreover, the consistent phenomena in HSV-1-infected A549 cells with IFI16^−/−^, indicating that IFI204 might facilitate cGAS-STING DNA sensing pathway that leads to IRF3 activation during the infection of HSV-1 [48]. Similarly, knockdown of IFI204 by small interfering RNA significantly inhibited IFN-β release in response to bacterial infections such as *Francisella novicida* [44], *Mycobacterium bovis* [49], *Staphylococcus aureus* [50], demonstrating IFI204 is essential for host defense against intracellular and extracellular bacterial infection.

IFI16, a sequence-independent nuclear innate sensor ALR, was also proposed to stimulate other cellular pathways upon its binding to viral DNA [40]. Several reports assert that DNA of *herpesviruses Kaposi’s sarcoma-associated herpesvirus* (KSHV), *Epstein-Barr virus* (EBV), and *herpes simplex virus 1* (HSV-1) during infection assembles an IFI16-containing oligomeric structure, leading to the production of active caspase-1 and IL-1β [51, 52]. Furthermore, during HSV-1 infection, IFI16 recognizes HIV-1 proviral DNA in nuclei of infected human foreskin fibroblasts (HEFs), inducing IFN-β production via the cytoplasmic STING-TBK1-IRF3 pathway [51, 53]. Besides, IFI16 was also reported to sense *Listeria monocytogenes* DNA in human macrophages, inducing IFN-β expression in a manner dependent on cGAS-STING [54].

### NLRP3 inflammasome regulates cGAS-STING

NLRP3 inflammasome is composed of the cytoplasmic sensor NLRP3, the adaptor ASC and the effector caspase-1. Elevated *p*-TBK1 and *p*-IRF3 in colonic tissues and enhanced IFN-β levels after NLRP3 deficiency were observed in the mice subjected to whole abdomen radiation by timed exposure to X-ray at a cumulative dose [55], suggesting that NLRP3 deficiency led to an increase in cGAS-STING-mediated IFN-β production by radiation. NLRP3 deficiency increased the production of *type I* IFN and enhanced the resistance of the host to Zika virus in vitro and in vivo [56], which unraveled a novel antagonistic mechanism by which Zika suppresses the host immune response by manipulating the interplay between inflammasome and *type I* IFN signaling, which might guide the rational design of therapeutics in the future.

### Caspases regulate cGAS-STING

Increased IFN production in response to DNA viral infection, but not RNA viral attack, was detected in the inflammatory response of Casp-1^−/−^ macrophages [57]. Caspase-1 interacted with cGAS during canonical and non-canonical inflammasome activation, cleaved cGAS and inhibited STING-mediated IFN production [57]. Upon inflammasome activation, caspase-1 binded directly to cGAS via its p20 domain and cleaved human cGAS at the D140/157 site, leading to a reduction in cGAMP production and cytokine expression. Also, caspase-4 and caspase-5 in humans and caspase-11 in mice cleaved cGAS in lipopolysaccharide (LPS)-induced activation of non-canonical inflammasome [57]. Consistently, induction of cGAS cleavage during Zika virus infection by caspase-1 inhibited phosphorylation of TBK1 and IRF3 and reduced *type I* IFN production, thereby evading the antiviral response [56]. In conclusion, canonical and non-canonical inflammasome activation induce the production of active caspase-1, which interacts with cGAS and in turn inhibits cGAS-STING-mediated *type I* IFN production (Fig. 1).

### GSDMD regulates cGAS-STING

The pore forming activity of GSDMD is located in gasdermin-N domain, while the gasdermin-C domain inhibits its pore forming activity. The release of gasdermin-N domain migrated to the cell membrane, formed pores with an inner diameter of 10–15 nm, thereby promoting pyroptosis [58–61]. Mice deficient in GSDMD exhibited an enhanced IFN-β response to *Francisella novicida* infection, and GSDMD negatively regulated the IFN-β response in a manner independent of pyroptosis and IL-1β [62]. GSDMD activated by AIM2 inflammasome depleted intracellular K^+^ through the membrane pores, which is sufficient and essential for the inhibition of the cGAS-dependent IFN-β response, and thereby inhibited the cGAS-driven *type I* IFN response to macrophage DNA and *F. novicida* infection [62]. In summary, the GSDMD-K^+^ efflux axis targets cGAS to reduce the synthesis of cGAMP, thereby inhibiting STING signaling and reducing IFN-β production (Fig. 1).

### The CARD domain of ASC regulates cGAS-STING

The ligand protein ASC consists of two domains, a PYD domain at the N-terminal and a CARD domain at the C-terminal. ASC recruits caspase-1 containing the CARD domain via CARD-CARD interactions to form inflammasome. ASC deficiency led to increased IFN production during DNA virus infection [57]. The CARD domain of ASC in AIM2 inflammasome was recently found to bind to the N-terminal domain of STING, thereby inhibiting the interaction of STING with TBK1 and thus negatively regulating the cGAS-STING signaling pathway [34]. NLRC3 protein containing the CARD domain blocked *type I* IFN response and IL-1β secretion by competing with ASC for caspase-1 binding, disrupting ASC speck formation, and interfering with NLRP3 inflammasome assembly and activation [63]. ASC in myeloid-derived macrophages and dendritic cells inhibited the interaction of STING with downstream TBK1, thereby reducing the induction of *type I* IFN [34]. Interestingly, a negative correlation between ASC expression and IFN-β levels was also observed in tuberculosis patients [34]. In summary, the CARD domain of ASC is essential for regulating the cGAS-STING signaling pathway (Fig. 1).

### Nod-like acceptors (NLRs) regulate cGAS-STING

Except as described above, there are various other inflammasomes, such as NLRX1, NLRP2, NLRC3, NLRC4, NLRC5, NLRP6, NLRP12 [64–70]. Recent studies have shown the emerging roles of NLRs in the cGAS-STING signaling pathway. Most NLRs positively influence inflammatory responses, particularly the inflammasome NLRs. However, emerging studies have revealed that NLRC3 negatively affect *type I* IFN response by sequestering and attenuating STING activation [63, 67, 68]. NLRC3 binds viral DNA and other nucleic acids via its LRR domain, which enhances the ATPase activity of nucleic acids. Furthermore, the ATP binding by NLRC3 reduces its interaction with STING, resulting in decreased production of IFN-β and IL-6 [67, 68]. NLRC3 also interacts with pro-caspase 1 and ASC through its CARD domain, thereby preventing the formation of NLRP3 and NLRC4 inflammasomes and further inhibiting cell pyroptosis [63]. Similar to NLRC3, NLRX1 interacts with STING through its nucleotide-binding domain (NBD), which results in a block of STING-TBK1 interaction thereby inhibiting TBK1 activation required for *type I* IFN production [69]. NLRP2 directly interacts with TBK1, disrupting the TBK1-IRF3 interaction and interfering with TBK1-induced IRF3 phosphorylation, thereby inhibiting IFN signaling [70]. NLRP4 negatively modulates *type I* IFN signal transduction through activation of TBK1, which is degraded by K48-associated ubiquitination by the E3 ubiquitin ligase DTX4 [71]. NLRP11 limits *type I* IFN activation by impairing TBK1-induced IFN-β promoter activity, suggesting its potential involvement in the cGAS-STING signaling pathway [72]. NLRP14 physically interacted with STING components and facilitated the ubiquitination and degradation of TBK1, which mediated the interactions and inhibitory function [73]. NLRP6 binds viral RNA via RNA helicase Dhx15 and interacts with MAVS (mitochondrial anti-viral signaling) to trigger the production of *type I* IFN [74]. NLRC4 promotes the cGAS-STING pathway by enhancing TBK1 interaction with the E3 ubiquitin ligase CBL to promote K63-linked polyubiquitination and subsequent activation of TBK1 [75, 76]. Furthermore, NLRC5 has the ability to stimulate the production of *type I* IFN and pro-inflammatory cytokines by fibroblasts and primary human cells when infected with cytomegalovirus or Sendai virus [77, 78].

## cGAS-STING regulates inflammasomes and pyroptosis

### cGAS-STING regulates NLRP3 inflammasome and pyroptosis

**Fig. 2:**
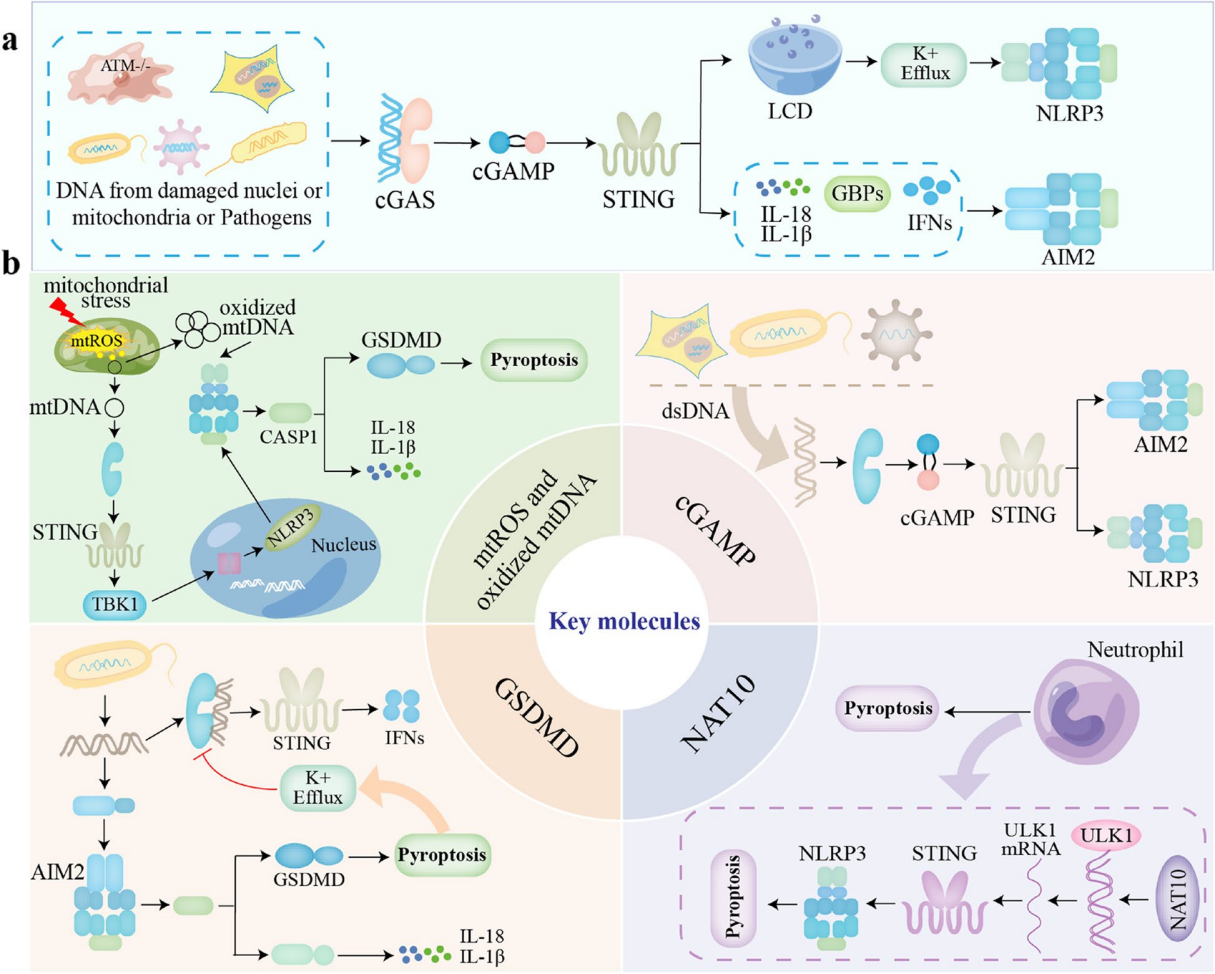
cGAS-STING regulates inflammasomes and pyroptosis, and the key molecules in the crosstalk network of cGAS-STING, inflammasomes, and pyroptosis (**a**) cGAS-STING regulates AIM2 inflammasome, NLRP3 inflammasome, and pyroptosis. (**b**) the key molecules, including ox-mtDNA, mtROS, GSDMD, NAT10, ULK1, and cGAMP, in the crosstalk network of cGAS-STING, inflammasomes, and pyroptosis

The cGAS-STING-NLRP3 signaling pathway is a specific mechanism that facilitates the activation of the NLRP3 inflammasome and the secretion of IL-1β in response to DNA virus infection and cytoplasmic DNA stimulation (Fig. 2). In human myeloid cells, the cGAS-STING pathway was necessary for cytoplasmic DNA-induced NLRP3 activation during viral and bacterial infection [79]; similarly, studies have shown that the STING-NLRP3 axis is critical for the pro-inflammatory response induced by *Chlamydia trachomatis* and aged macrophages [80, 81]. Furthermore, STING-IRF3 could trigger LPS-induced cardiac dysfunction, inflammation and pyroptosis by activating NLRP3 in mice [82]. In addition, in septic mouse neutrophils, downregulation of NAT10 inhibited ULK1 expression, activated the cGAS-STING pathway, induced NLRP3 inflammasome activation, and thus promoted neutrophil pyroptosis [83]. Moreover, the cGAS-STING pathway was activated in myelodysplastic syndromes (MDS) to induce IFN-stimulated genes (ISG), which triggered the activation of NLRP3 inflammasome [84].

During viral and bacterial infections in human myeloid cells, NLRP3 was tightly linked to the upstream cGAS-STING pathway, inducing NLRP3 inflammasome activation and coordinating lysosomal cell death (LCD) in a K^+^ efflux-dependent manner [79]. Cytoplasmic DNA was recognized by cGAS, and then STING was activated and transported to the lysosome, triggering membrane permeation and causing LCD [85]. Lysosomal lysed cathepsin leaked into the cytoplasm, altered plasma membrane permeability, activated K^+^ efflux upstream of NLRP3 and ultimately induced pyroptosis [79], which triggered a series of inflammatory cascade responses. In summary, DNA-triggered the NLRP3 inflammasome activation is dependent on the cGAS-STING-LCD axis, and targeting this pathway would ameliorate the inflammatory response associated with cytoplasmic DNA receptor evocation.

Available studies indicate that STING interaction with NLRP3 in response to cytoplasmic DNA stimulation promotes NLRP3 inflammasome activation in several ways. Firstly, STING recruited NLRP3 to promote its localization in the ER, thereby promoting the formation of NLRP3 inflammasome [86]. Secondly, TM5 (151-160aa) of STING interacted with NACHT and LRR domain in NLRP3 to attenuate NLRP3 polyubiquitination associated with K48 and K63, i.e., STING deubiquitinated NLRP3 to activate the NLRP3 inflammasome [86]. Thirdly, in an epistatic regulatory mechanism study, H3K4-specific histone methyltransferase WDR5 and H3K79 methyltransferase DOT1L inhibitors were found to significantly reduce STING overexpression-mediated NLRP3 upregulation, suggesting that STING promoted NLRP3 promoter region histone methylation via WDR5/DOT1L, thereby recruiting IRF3 to increase NLRP3 transcription [87].

### cGAS-STING regulates AIM2 inflammasome

AIM2 is the only member of the PYHIN gene family that is truly homologous between mouse and human [88], and gain-of-function and loss-of-function studies at the cellular level have shown that human AIM2 functions in the same way as its mouse counterpart, AIM2 [89]. Thus, studies of AIM2 in the mouse system can be extrapolated to humans. AIM2, an innate sensor of the canarypox virus vector ALVAC, triggers inflammasome activation in human and mouse antigen-presenting cells. CRISPR/Cas9 analysis reveals that ALVAC activated the AIM2 inflammasome through stimulation of the cGAS-IFI16-STING-type I IFN pathway [90]. Cytoplasmic DNA in ataxia-telangiectasia mutated (ATM)-deficient microglia was sensed by cGAS, thereby activating the cGAS-STING pathway to initiate an antiviral response, and triggering the activation of the AIM2 inflammasome [91]. Activation of the STING pathway during *Francisella* infection promoted *type I* IFN production and IRF1 expression, which induced guanylate-binding proteins (GBPs) targeting bacterial vesicles to disrupt their membranes, allowing bacterial products to be sensed by AIM2 and subsequently activating the AIM2 inflammasome [92]. The STING-dependent *type I* IFN signaling pathway was essential for the GBP-mediated release of *Brucella* DNA into the cytosol and the subsequent activation of AIM2 [93]. Collectively, these data indicated that the STING signaling axis-induced *type I* IFN is necessary for therelease of cytoplasmic DNA for activation of the AIM2 inflammasome (Fig. 2).

*Chlamydia trachomatis* replication or metabolism induced *type I* interferon responses are critical mediators of inflammasome activation and pyroptosis in macrophages [80]. cGAS-STING-dependent TNF and IFN signaling triggers necroptosis in response to cytosolic DNA [94]. In addition, mtDNA activates the STING pathway that subsequently enhances RIPK3/MLKL expression to trigger necroptosis [95]. Emerging evidence suggests that ER stress associated with STING activation can trigger apoptosis [96–98]. Therefore, cGAS-STING signaling can trigger multiple cell death pathways including pyroptosis, apoptosis, and necrosis, and a better understanding of the regulatory mechanisms across different cell types, states and health, and environmental and/or stimulus-dependent mechanisms will require further investigation. In addition, the key role played by the cGAS-STING signaling pathway in multiple cell death pathways, such as the newly described PANopoptosis, ferroptosis, and cuproptosis, remains to be thoroughly investigated and explored. Further mechanistic elucidation will help answer questions such as what determines the bidirectional regulation of cGAS-STING and cell death pathways. More importantly, the answers to these critical questions will provide new ways and methods to target the cGAS-STING-mediated cell death pathways for the treatment of infectious diseases, inflammatory diseases, and so on.

## Key molecules in the crosstalk network of cGAS-STING, inflammasomes, and pyroptosis

### Ox-mtDNA and mtROS

Mitochondria regulates the innate immune system through the release of numerous pro-inflammatory signals, such as mitochondrial reactive oxygen species (mtROS), mitochondrial DNA (mtDNA) and Ca^2+^, which is vital for the inflammasomes and cGAS-STING pathways activation (Fig. 2) [99–102]. Exposure of newly synthesized mtDNA to ROS induces oxidized mtDNA (Ox-mtDNA) production [103]. Ox-mtDNA was either repaired by 8-oxoguanine-DNA glycosylase (OGG1) or cleaved into 500–650 bp fragments by flap-structure-specific endonuclease 1 (FEN1). These fragments leaked from the mitochondria via 1-methyl-4-phenyl-1,2,3,6-tetrahydropyridine (mPTP)- and voltage-dependent anion channel (VDAC)-dependent channels and triggered NLRP3 inflammasome activation in the cytoplasm [104]. Ox-mtDNA fragments also led to phosphorylation of the STING Ser365 site, which was required for cGAS-STING-IRF3 binding and activation of the *type I* IFN response [104]. Brown adipose tissue (BAT) acts as an important thermogenic organ, regulating energy metabolism through thermogenesis [105–107]. BAT inflammation is associated with mitochondrial dysfunction and impaired thermogenesis [108–110]. mtROS was scavenged by mitochondrial thioredoxin-2 (TRX2), and TRX2 deficiency induced massive mtROS production, mitochondrial integrity disruption, and cytosolic release of mtDNA, which activated aberrant innate immune responses in BAT, including the cGAS-STING and the NLRP3 inflammasome pathways [111].

XBP1 deficiency induced the excessive production of ROS to promote hepatocyte pyroptosis through the activation of NLRP3 and pyroptosis signaling, which made it easier to release the mtDNA into the extracellular space. mtDNA released from thioacetamide (TAA)-stressed hepatocytes was engulfed by macrophages, further inducing cGAS- and dose-dependent macrophage STING activation [112]. The mitochondrial oxidative stress response also plays a role in bacterial infection. Mitochondrial oxidative stress-induced release of mtDNA in bacterial infection mediated the secretion of *type I* IFNs via the cGAS-STING pathway and triggered activation of the NLRP3 inflammasome [113, 114]. *Mycobacterium abscessus* facilitated the production of Ox-mtDNA to enhance cGAS-STING-dependent IFN production and NLRP3 inflammasome-mediated IL-1β [115]. Intracellular mtROS/mtDNA induced bacterial replication after phagosome rupture and escaped into the cytoplasm, disrupting membrane integrity in a *type I* IFN-dependent manner [115]. *Type I* IFN, on the other hand, inhibited NLRP3 inflammasome activation via the STAT pathway [116, 117]. In addition, *type I* IFN-mediated generation of nitric oxide synthase (iNOS) and NO inhibited NLRP3 protein oligomerization, thereby preventing the assembly of NLRP3 inflammasome [118].

### GSDMD

GSDMD not only promotes the effective release of IL-1β and IL-18, but also acts as an end-effector of pyroptosis. Another function of GSDMD is to promote the non-selective release of K^+^ in cells. During infection with *F. novicida*, cGAS-induced IFNs were inhibited by GSDMD-mediated K^+^ efflux, and GSDMD deficiency was found to prevent cytoplasmic K^+^ efflux and enhance dsDNA binding to cGAS, thereby activating the cGAS-STING pathway and promoting IFNs secretion [62], suggesting that GSDMD inhibits cGAS mediated IFNs secretion. Furthermore, given the central role of the cGAS pathway in the innate immune response, it is expected that various modulations and modifications to cGAS control its activity. Further studies indicates that members of the tripartite motif 56 (TRIM56) induced the Lys335 monoubiquitination of cGAS, which resulted in a marked increase of its dimerization, DNA-binding activity, and cGAMP production of cGAS [119, 120]. In summary, bacterial dsDNA triggers the activation of inflammasomes, leads to GSDMD cleavage, and causes K^+^ efflux, thereby limiting the binding of bacterial dsDNA to cGAS, inhibiting the activation of the cGAS-STING pathway, and disrupting the inflammatory response of IFNs (Fig. 2).

### NAT10 and ULK1

As the first identified RNA acetyltransferase, N-acetyltransferase 10 (NAT10) catalyzes the N4 acetylation of cytidine (ac4C) to regulate mRNA stability and translation, and is implicated in a variety of cellular processes including cell division, cellular senescence, autophagy and DNA damage [121, 122]. Neutrophils play an important role in the progression of sepsis as major effector cells against infection and as important regulators of innate immunity [123]. During sepsis, large amounts of bacterial products (e.g., CpG DNA) as well as the host’s own DNA (including nuclear and mitochondrial DNA) were released into the cytoplasm, leading to the activation of cGAS-STING and pyroptosis [124]. NAT10 was a negative regulator of neutrophil pyroptosis, and its reduced expression led to increased neutrophil pyroptosis and secretion of large amounts of the pro-inflammatory cytokines IL-1β and IL-18 [83]. In neutrophil, down-regulation of NAT10 led to a decrease in UNC-52-like kinase 1 (ULK1) expression level. In contrast, as a regulator of STING phosphorylation, deletion of ULK1 activated the STING-IRF3 pathway, which subsequently triggered NLRP3 inflammasome activation and neutrophil pyroptosis [125]. On the other hand, ULK1 has been shown to be involved in NLRP3 autophagy, suggesting that ULK1 has a direct regulatory effect on the NLRP3 inflammasome in addition to inhibiting STING (Fig. 2) [126].

### cGAMP

cGAMP, as a second messenger, directly binds to STING and its upstream key synthetase cGAS, which further activated TBK1, induced IRF3 and NF-κB into the nucleus, produced *type I* IFN and cytokines, and defended against various viral infections. Studies showed that cGAMP increased the activation of AIM2 and NLRP3 inflammasomes via cGAS-STING (Fig. 2). cGAMP induced the activation of AIM2 and NLRP3 inflammasomes in addition to *type I* IFN by increasing mRNAs encoding key components of the inflammasome (AIM2, NLRP3, Casp1, IL-1β, and ASC), thereby inhibiting DNA virus infection [127].

## Diseases induced by the crosstalk network of cGAS-STING, inflammasomes, and pyroptosis

A well-coordinated immune response is essential for recognizing and eliminating threats from foreign substances and tissue damage. However, uncontrolled inflammation can contribute to the pathology of chronic inflammatory and degenerative diseases, as well as cancer. Chronic inflammation plays a prominent role in driving carcinogenesis, as various chronic inflammatory conditions are associated with an increased risk of cancer. This leads to the accumulation of DNA damage and production of local inflammatory cytokines. Eventually, the phenotype shifts towards an altered homeostasis and becomes irreversibly responsive to continued inflammation, resulting in malignancy [128–130]. Recent studies support the idea that inflammation influences the fate of various components within the complex tumor microenvironment, ultimately creating a tumor-promoting environment through reciprocal communication that promotes carcinogenesis either through direct mutagenesis or by activating cytokine responses that effectively shape the host response [128, 131].

**Fig. 3:**
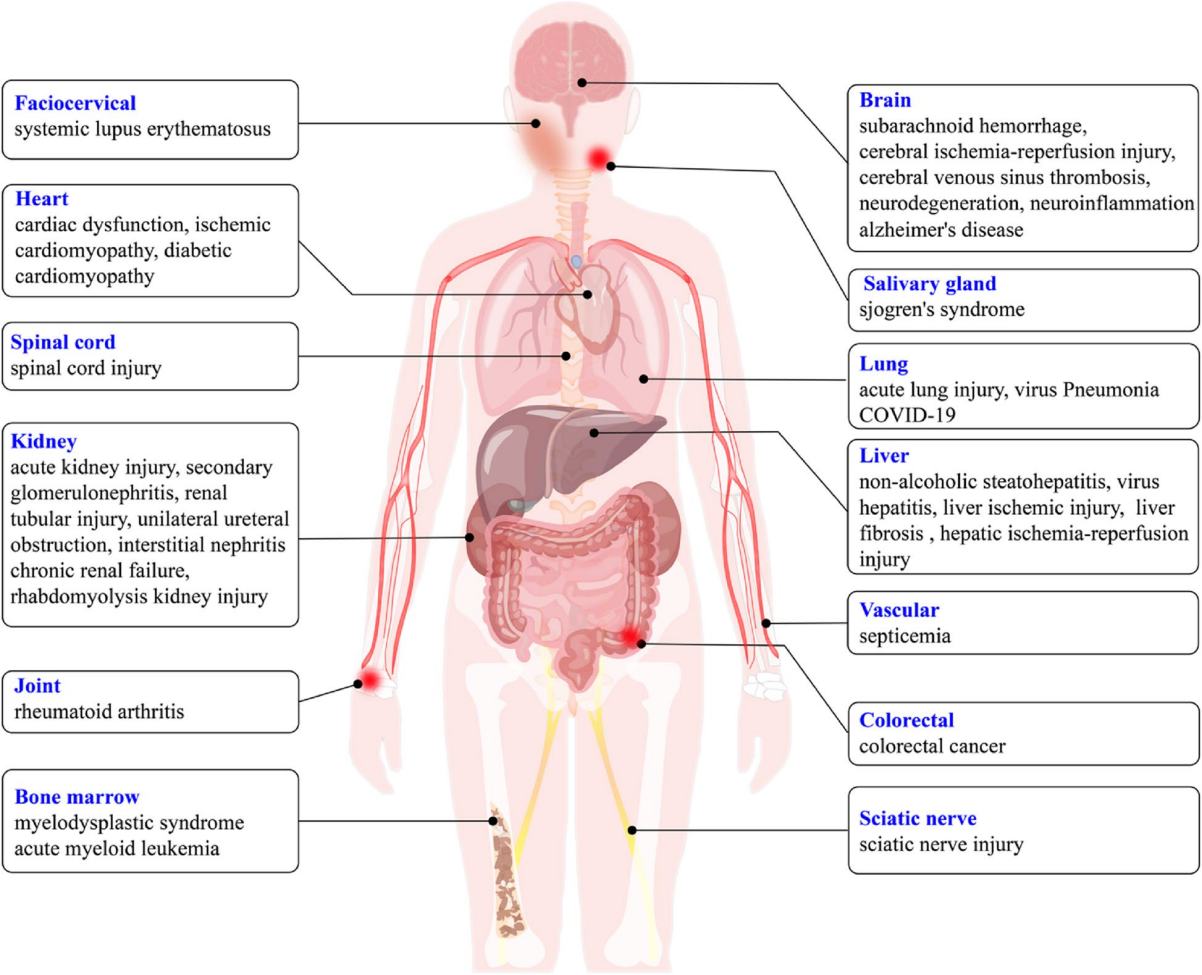
Diseases induced by the crosstalk network of cGAS-STING, inflammasomes, and pyroptosis. Evidence shows that the crosstalk network of cGAS-STING, inflammasomes, and pyroptosis is involved in the pathogenesis of a number of diseases, such as lung diseases, liver diseases, kidney diseases, cardiac dysfunction, spinal injury, arthritis, nervous system diseases, autoimmune diseases, and malignant tumors

There is increasing evidence that the risk of developing chronic inflammation can be traced back to early development, and its consequences are now known to extend throughout the life span, affecting health and mortality risk in adulthood [132–134]. Therefore, the “inflammatory fire” sparked by the host response requires tight management to avoid spreading and causing irreversible damage. Recent evidence has demonstrated that activation of the cGAS-STING axis in response to cytosolic DNA stimulation engaged in inflammasome activation [79, 86] and GSDMD-triggered pyroptosis [135], which is characterized by the dysfunctions of the immune system and the aberrant secretion of inflammatory cytokine. As a result, the interplay among the cGAS-STING axis, inflammasome, and pyroptosis builds a wide range of important monitoring systems in response to tissue damage and pathogen invasion. Abnormalities of this crosstalk cause a variety of human diseases, including infectious diseases, autoimmune diseases, tumors, organ fibrosis and neurodegenerative diseases [11, 136–138]. In view of the critical role of cGAS-STING, inflammasomes and pyroptosis in immune and inflammatory responses, we then focused on the related diseases induced by this crosstalk network with the aim of providing clues for their prevention and treatment (Fig. 3).

### Cardiac dysfunction

cGAS-STING pathway can activate the NLRP3 inflammasome, thereby exacerbating inflammation in the myocardium and promoting cardiac dysfunction. In cardiomyocytes, STING binds to IRF3 and phosphorylates IRF3, which subsequently translocated into nucleus and increased the expression of NLRP3 [82]. In contrast, STING knockdown inhibited IRF3 phosphorylation and perinuclear translocation, thereby suppressing NLRP3-mediated cardiomyocyte inflammation and pyroptosis, improving cardiac function and increasing survival [82]. Also, in diabetic cardiomyopathy (DCM), the production of free fatty acids induced oxidative mitochondrial damage, activated the cGAS-STING and NLRP3 inflammasome signaling pathways, and ultimately promoted myocardial hypertrophy in DCM by promoting cardiomyocyte pyroptosis [139]. Activation of STING enhanced GSDMD-mediated cardiac hypertrophy [140]. Consistently, knock down cardiomyocyte STING in DCM attenuated cardiac pyroptosis and inflammatory responses, suppressed DCM-induced cardiac hypertrophy, and restored cardiac function [139]. Therefore, targeting cardiomyocyte STING, NLRP3 inflammasome, and pyroptosis may be a potential therapeutic strategy to prevent cardiomyopathy.

### Acute lung injury (ALI)

Macrophages are the most abundant immune cells in lung tissue, and inhibition of inflammatory signaling pathways in macrophages is essential to maintain tissue homeostasis. Macrophages are more likely to exhibit cellular senescence, impaired mitochondria, and abnormal activation of the cGAS-STING and NLRP3 inflammasome pathways, which predispose mice to severe viral pneumonia during infection [141]. Cytoplasmic mtDNA and STING transcription factor (c-Myc) synergistically activated the cGAS-STING pathway in LPS-induced ALI, which subsequently exacerbated ALI inflammation by triggering NLRP3 inflammasome activation and pyroptosis [142, 143]. The STING agonist diamidobenzimidazole (diABZI), was internalized into the cytoplasm and induced STING activation and dimerization, and upregulated apoptosis, pyroptosis and necroptosis (PANoptosis), which enhanced lung inflammation with severe acute respiratory distress syndrome [144]. Radiation therapy-induced self-dsDNA was leaked into the bronchoalveolar space and subsequently triggered cGAS-STING activation and downstream NLRP3-mediated pyroptosis, providing a mechanistic basis for pyroptosis that connects cGAS-STING activation to the exacerbation of initial radiation-induced lung injury [145]. In summary, the cytoplasmic cGAS-STING-NLRP3 pathways contribute to LPS-induced ALI. Based on these findings, targeting the cytoplasmic cGAS-STING-NLRP3 pathways may be a therapeutic target for ALI.

### Liver diseases

The liver is a prime target for toxins and acute injury as the primary organ for removal of various drugs and foreign pathogens. Macrophage infiltration is a characteristic of liver inflammation, and macrophage activation of the cGAS-STING and inflammasome pathways are important drivers of numerous liver diseases [146–149]. Increased STING activation was observed in human and mouse liver with nonalcoholic steatohepatitis [150, 151]. Macrophage STING activation in acute ischemic liver injury facilitated by mtDNA release from injured hepatocytes [81]. Ox-mtDNA produced under oxidative stress of liver injury triggered the activation of NLRP3 inflammasome [152]. Experimental CCl_4_ induced liver fibrosis and enhanced cGAS-STING activation in liver tissue, while STING deficiency attenuated liver inflammation and fibrosis [153–155]. RNA sequencing of livers from mice with CCl_4_-induced liver fibrosis revealed that the STING and NLRP3 inflammasome signaling pathways were activated during liver fibrosis, and the activation of these two pathways were also verified in human and mouse cirrhotic tissues [87]. STING and NLRP3 signaling pathways are activated in cirrhosis, and both knockdown of STING and STING inhibitor C-176 significantly inhibited NLRP3 expression and hepatocyte pyroptosis [87], suggesting that STING can induce hepatocyte pyroptosis through activation of the NLRP3 inflammasome.

GSDMD-mediated hepatocyte pyroptosis contributes to accelerated pathogenesis in acute and chronic liver disease [156–159]. In mice, activation of the NLRP3 inflammasome resulted in hepatocyte pyroptosis, hepatic inflammation, and liver fibrosis [158]. In addition, caspase-1 and GSDMD-mediated hepatocyte pyroptosis induced stellate cell activation through the release of inflammatory factors, thereby promoting the development of liver fibrosis [159]. STING induced hepatic ischemia-reperfusion injury (IRI) by promoting calcium-dependent caspase-1-GSDMD in macrophages, and STING expression enhanced with increased hepatic IRI, while knockdown of STING attenuated hepatic IRI [160]. ROS also plays a key role in hepatocyte pyroptosis [161, 162]. Upregulation of ROS levels promoted GSDMD cleavage, activated the GSDMD-N terminus, and induced cell membrane pore formation, thereby promoting pyroptosis [163, 164]. In addition, in TAA-induced liver injury, hepatocyte ROS-NLRP3-caspase-1-GSDMD activity was increased and hepatocyte pyroptosis was detected [112]. XBP1 deficiency in hepatocytes promoted ROS production to activate NLRP3-Caspase-1-GSDMD signaling, which promoted extracellular release of mtDNA and macrophage phagocytosis of mtDNA, further activated the cGAS-STING pathway, thereby promoting hepatocyte pyroptosis [112].

### Kidney diseases

Acute kidney injury (AKI) is marked by a progression of rapid loss of kidney function that can lead to chronic kidney disease (CKD) and end-stage renal disease (ESRD) [165, 166]. Recent studies suggest that mtDNA-associated chronic inflammatory responses are associated with the pathogenesis of AKI and the development of CKD [167–169]. Mitochondrial damage was induced in AKI, leading to leakage of mtDNA into the cytoplasm and activation of the cGAS-STING pathway, which phosphorylated TBK1 and IRF3, promoted the secretion of inflammatory factors and exacerbated the inflammatory response [168]. Activation of the cGAS-STING pathway was observed in multiple AKI mouse models and AKI patients [168, 170, 171]. STING knockout mice exhibited reduced renal function, tubular damage and inflammation after cisplatin treatment [168]. In addition, STING mediated secondary renal inflammation and tubular injury. STING and NLRP3 inflammasome pathways played important roles in unilateral ureteral obstruction, adenine-induced tubulointerstitial nephritis and chronic renal failure [172–174]. Expression of G2-type apolipoprotein APOL1 (G2 APOL1) in mouse kidney cells led to activation of cGAS-STING and NLRP3 inflammasome, and APOL1 expression correlated with caspase-1 and GSDMD levels [175]. In a RIAKI mouse model, although AIM2 deficiency inhibited renal macrophage pyroptosis, it surprisingly accentuated abnormal inflammation as evidenced by massive macrophage aggregation (CXCR3^+^CD206^+^) and activation of the cGAS-STING-TBK1-IRF3 pathway, which subsequently promoted maturation and secretion of pro-inflammatory cytokines. Meanwhile, dsDNA-induced AIM2-deficient cells escaped rapid pyroptotic elimination and participated in STING-TBK1-IRF3/NF-κB pathways, leading to an exacerbation of the inflammatory phenotypes [33]. These finding suggested that the rapid macrophage cell death induced by dsDNA may serve as an anti-inflammatory program and may determine the healing process of RIAKI.

### Nervous system inflammation

Microglia are important mediators of neuroinflammation and immune response after CNS injury [176, 177]. NLRP3 inflammasome-mediated microglia pyroptosis is associated with the pathogenesis of subarachnoid hemorrhage [178], cerebral ischemia/reperfusion injury [179, 180], and spinal cord injury [181]. Recent studies have shown that cytoplasmic DNA induces NLRP3 and AIM2 inflammasomes activation and GSDMD-triggered microglia pyroptosis through activation of the cGAS-STING pathway [79, 86, 91, 135]. Importantly, elevation in cGAS and STING occurred mainly in microglia in damaged cortex after cerebral venous sinus thrombosis (CVST), and the same cellular localization was reported in cerebral ischemia/reperfusion (I/R) [135] and subarachnoid hemorrhage models [5]. Accumulation of dsDNA on cell membranes triggered activation of cGAS-STING pathway in intracranial venous and CVST, which subsequently induced NLRP3 inflammasome activation, microglia pyroptosis, and increased the neuroinflammatory burden [182]. Hyperphosphorylated Tau in the brain is an important pathological feature of patients with neurodegenerative diseases. Tau induced NLRP3 inflammasome activation, which drived tau hyperphosphorylation and exacerbated neuroinflammation, and the biological process may be attributed to the immune stimulating activity, especially the cGAS-STING pathway [183–185]. The STING agonist CMA significant increased STING expression in microglia after subarachnoid hemorrhage (SAH) and exacerbation of neuronal damage [5]. In addition, in the brains of patients with different neurodegenerative diseases, serum/glucocorticoid-related kinase 1 (SGK1) was elevated. SGK1 expression is widely detected in the brain, and it is increased in pathologic conditions such as Rett syndrome [186], Alzheimer disease (AD) [187, 188], multiple sclerosis [189], amyotrophic lateral sclerosis [190], and neuropathic pain [191], collectively suggesting that SGK1 plays pathogenic roles in neurodegenerative disorders. Inhibition of glial SGK1 corrects the pro-inflammatory characteristics of glia by reducing intracellular NF-κB, NLRP3 inflammasome and cGAS-STING mediated inflammatory pathways [192]. Activation of the cGAS-STING pathway in AD mice triggered the formation of NLRP3 inflammasome, exacerbated cellular senescence and inflammatory responses, and nicotinamide riboside (NR) treatment exerted beneficial effects through the cGAS-STING pathway [193]. Furthermore, inflammatory response-induced microglia activation was associated with neurological deficits after traumatic brain injury (TBI). In contrast, microglia cGAS-STING activation promoted neuroinflammatory responses after TBI, in part through activation of the NLRP3 inflammasome [194]. In conclusion, the cGAS-STING-NLRP3 signaling pathway may serve as a potential therapeutic target for neuroinflammation-induced neurological dysfunction.

### Myelodysplastic syndrome (MDSs) and spinal injury (SCI)

NLRP3 inflammasome, pyroptosis and cGAS-STING contribute to neuroinflammation in myelodysplastic syndromes (MDSs) and spinal cord injury (SCI) [84, 195]. cGAS-STING induced activation of interferon-stimulating factor (ISG), triggered NLRP3 inflammasome activation, and exacerbated bone marrow injury [84]. Further studies revealed that caspase-1 degraded the erythroid transcription factor GATA-binding protein 1, triggering anemia and myeloid bias to exacerbate the injury [84]. MDSs hematopoietic stem and progenitor cells (HSPCs) overexpressed inflammasome proteins and exhibited NLRP3 inflammasomes activation that directly produced IL-1β and IL-18, and drived pyroptosis [196]. As with somatic mutations, excess alarm protein S100A9 in bone marrow plasma activated NADPH oxidase (NOX), increased ROS levels, exposed cytoplasmic DNA to the cGAS-STING-NLRP3 axis, and promoted pyroptosis [196]. In addition, cGAS-STING and NLRP3 inflammasome activation in spinal microglia after sciatic nerve injury have been shown to exacerbate neuroinflammation in mice [195]. cGAS, STING, and NLRP3 were correlated with the extent of intervertebral disc degeneration by magnetic resonance imaging (MRI) and histopathology. Oxidative stress initiated the STING-dependent activation of the cGAS-STING axis and NLRP3-inflammasome-mediated pyroptosis in human nucleus pulposus cells [197]. Taken together, these data implicate the essential role of the cGAS-STING-NLRP3 axis and pyroptosis in the development of IVD degeneration and offer a potential treatment approach for the management of discogenic low back pain.

### Autoimmune diseases

Activating the cGAS-STING pathway confers host immunity and contributes to eliminating multiple pathogens, including viruses and bacteria. Meanwhile, excessive STING and inflammasome activation have been identified as contributing to the progression of autoinflammatory diseases such as systemic lupus erythematosus (SLE), rheumatoid arthritis (RA), acute myeloid leukemia (AML), sepsis and dry syndrome [83, 195, 198–202]. During SLE, STING and NLRP3 inflammasome activation mediated caspase-1 activation and promoted maturation and secretion of inflammatory factors [198, 199]. In addition, monocytes in SLE patients showed considerable activation of caspase-1 [200]. In acute myeloid leukemia (AML) with TP53 mutations, the therapeutic agent DNA methyltransferase inhibitors (DNMTis) expressed endogenous retroviruses (ERVs), IFNs and activated NLRP3 inflammasome in a STING-dependent manner [201]. DNA polymerase β (Pol β) was significantly decreased in peripheral blood mononuclear cells (PBMCs) of RA patients and mice with collagen-induced arthritis (CIA). Further studies revealed that Pol β knockdown led to DNA damage accumulation and cell membrane dsDNA leakage, which activated the cGAS-STING-IRF3-NF-κB signaling pathways and promoted pyroptosis [202].

### Malignant tumors

Growing evidence indicates that the innate immune response is critical to tumorigenesis and antitumor therapy [130, 203]. In mouse models and clinical patients, activation of the cGAS-STING pathway has been proven to reduce tumor growth and improve immunogenicity [204]. STING enhanced IL-18 and IL-1β generation by macrophages by activation of NLRP3, and IL-18 and IL-1β induced 4-1BBL and 4-1BB expression in macrophages and NK cells, respectively, which facilitated macrophage STING signaling to improve anti-tumor function, thus suppressing colorectal cancer liver metastasis [205]. Macrophage STING signaling pathway promoted NLRP3 inflammasome activation, enhanced anti-tumor function of NK cells, and inhibited liver metastasis from colorectal cancer [206]. However, cGAS-STING activation-mediated chronic inflammation can also promote tumor metastasis through the induction of immunosuppressive TME [9]. Cancer cell-produced cGAMP enhanced tumor growth and chemoresistance through activation of astrocyte STING and production of inflammatory cytokines [207].

### COVID-19

Severe COVID-19 is characterized by an excessive inflammatory response, including large cytokine expression, that involves a wide range of immune cells, including macrophages and neutrophils, that sense pathogens and damaged autologous structures and subsequently induce the production of inflammatory mediators. Infection and replication of SARS-CoV-2 in immune cells within the lung is a key driver of the disease. Inflammasome activation and the accompanying inflammatory response are necessary for lung inflammation in COVID-19 [208–211]. The cGAS-STING pathway, which controls immunity to cytosolic DNA, is a critical driver of aberrant* type I *IFN responses in COVID-19 [212, 213]. SARS-CoV-2 infection has a dual-edged sword effect on STING signaling, relying on the progressive stage of the disease and the infected tissue. Therefore, STING agonists or inhibitors are promising for the prevention and treatment of SARS-CoV-2. For example, STING agonists are used in the early stage of infection to activate the immune response in the body to kill the virus and inhibit its replication, and STING inhibitors are used in the middle and late stages of infection to reduce the excessive immune response of the body and reduce lung inflammation [212–216]. However, the specific application of STING modulators in the prevention and treatment of COVID-19 still needs further research, including the specific timing of administration and medication standards.

Although the interaction network between the inflammasome and cGAS-STING pathways has not been reported during COVID-19 infection, we have reasons to believe that there is an inseparable close relationship and feedback regulatory mechanism between the two. On the one hand, SARS-CoV-2 infection induces inflammasome activation and triggers multiple cell death pathways including pyroptosis, apoptosis, and necrosis, which may lead to the release of dsDNA in the nucleus and mitochondria into the cytoplasm under certain conditions. cGAS recognizes dsDNA without sequence difference and activates STING pathway to generate immune response. The continuous activation of the two pathways induces the production of a large number of inflammatory factors and aggravates the immune inflammatory response of the body. In addition, as mentioned in section 2, AIM2 inflammasome, AIM2-like receptors, NLRP3, caspases, GSDMD, and CARD domain of ASC can all participate in regulating the activition of cGAS-STING signaling pathway. It can be seen that the crosstalk of inflammasome and cGAS-STING pathways has not yet been clarified in COVID-19 patients, and how the two interact and how to regulate the body’s immunity are still key issues to be solved.

## Regulators of the crosstalk network of cGAS-STING, inflammasome, and pyroptosis

**Fig. 4:**
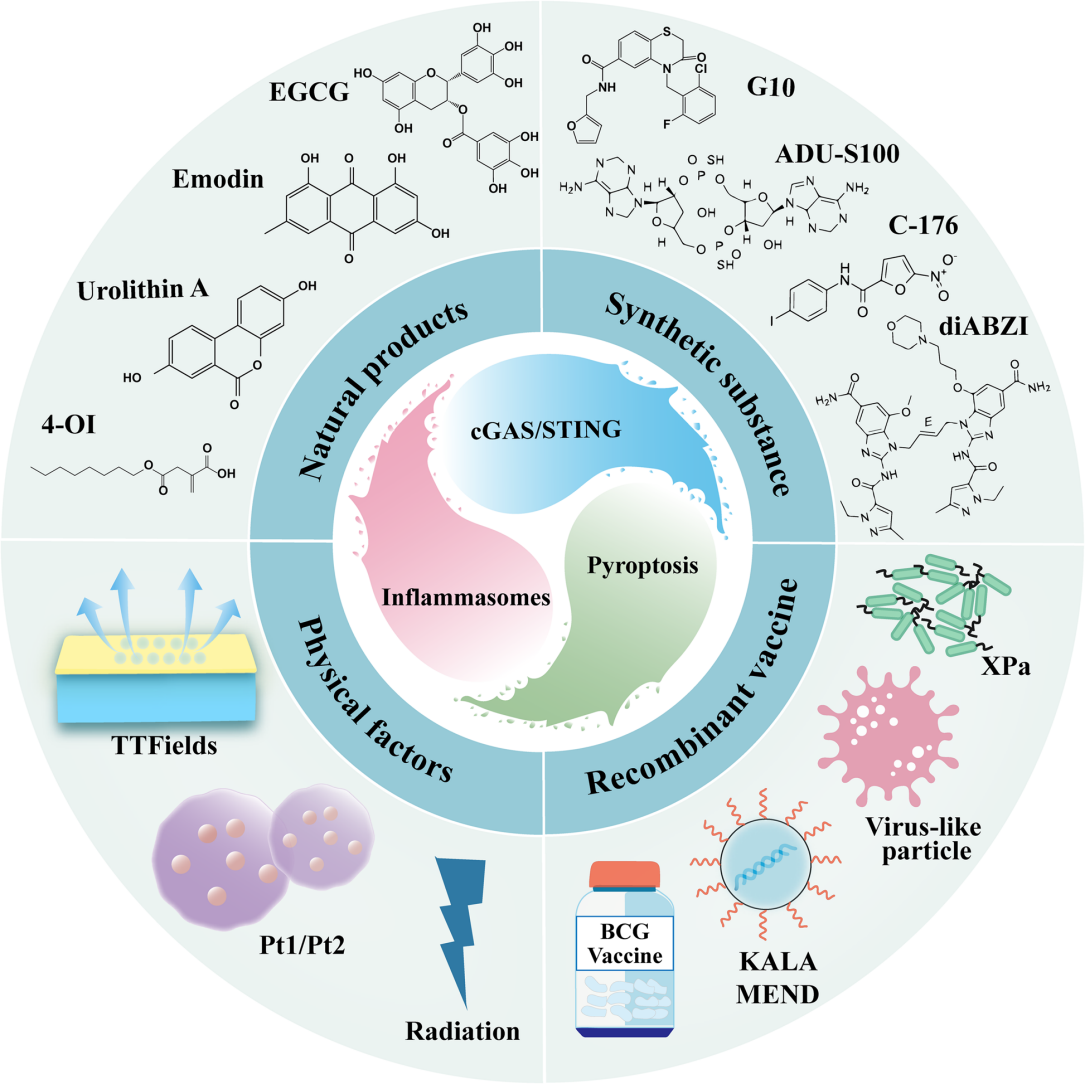
Regulators of the crosstalk network of cGAS-STING, inflammasome, and pyroptosis The involvement of natural products (such as EGCG, emodin, urolithin A, and 4-Octylic acid), synthetic substances (such as G10, diABZI, ADU-S100, and C-176), recombinant vaccines (such as Xpa, BCG vaccine, KALA MEND, and virus-like particle), and physical factors (such as TTFields, Pt1/Pt2, and Radiation) in regulating the cGAS-STING, inflammasomes and pyroptosis pathways crosstalk network, providing potential candidates for the treatment of related diseases

As described above the crosstalk network of cGAS-STING, inflammasome and pyroptosis is correlated with an elevated risk of the development of a broad range of chronic diseases that are currently the leading cause of morbidity and mortality throughout the world and are responsible for an enormous amount of human suffering. At the same time, the discovery of regulators such as agonists, inhibitors, vaccines and physical factors that could be explored to enrich this work and convert this work into meaningful strategies for improving human health (Fig. 4).

### Natural products

The cGAS-STING, inflammasome and pyroptosis pathways exacerbate the progression and course of various diseases through the crosstalk network, and therefore the search for their modulators is of great importance for disease prevention, treatment and recovery. Natural products are currently becoming an important source of drug discovery for disease treatment due to their broad pharmacological activity, high safety profile and diversity of targets.

4-Octylic acid (4-OI), an immunomodulatory derivative accumulated during macrophage activation, has attracted widespread attention for its anti-inflammatory and antioxidant properties. In vitro and in vivo experiments have shown that 4-OI inhibited the activation of the cGAS-STING-IRF3 pathway by eliminating mtROS production and mtDNA leakage in alveolar macrophages under oxidative stress, while alleviated LPS-induced NLRP3 inflammasome-mediated pyroptosis, which in turn ameliorated acute respiratory distress syndrome (ARDS) [217]. Epigallocatechin gallate (EGCG) is a catechin monomer isolated from tea and is a major component of green tea polyphenols. Advanced in vitro study that EGCG could block the activation of NLRP3 inflammasome through down-regulation of cGAS-STING-IRF3 pathway, and thus had significant protective effects against H_2_O_2_-induced apoptosis and inflammation in myeloid cells [218].

Several studies have shown that the physiologic concentration of hydrogen sulfide (H_2_S) has a vital role in the cardiovascular system through the regulation of biological functions and the maintenance of homeostasis in the body [219, 220]. Conversely, the lack of endogenous H_2_S is harmful and may lead to the development of various cardiovascular diseases, including atherosclerosis, hypertension, myocardial infarction and heart failure [221–223]. A high-choline diet reduced plasma H_2_S levels and induced cardiac dysfunction via the cGAS-STING-NLRP3 inflammasome pathways, while H_2_S treatment inhibited NLRP3 inflammasome activation mediated by cGAS-STING pathway activation, thereby restoring cardiac function [224]. As above discussed, the part of pathophysiological and pharmacological effects of H_2_S have been demonstrated in vitro and in vivo studies as well as in clinical disease. However, accounting for these pathophysiological responses will not be easy in preclinical models of disease. Emodin is a natural bioactive compound from herbal medicine with anti-inflammatory, antioxidant, anticancer, hepatoprotective and neuroprotective effects. In vivo and in vitro studies showed that emodin protected hepatocytes from acetaminophen (APAP)-induced liver injury by upregulating Nrf2-mediated antioxidant stress response, inhibiting NLRP3 inflammasome and cGAS-STING-IRF3 pathways [225]. Urolithin A, one of the principal intestinal metabolites of ellagitannins, attenuated fructose-induced hyperuricemic nephropathy through the promotion of Parkin-dependent mitophagy, thus limiting the inflammatory response mediated by the STING-NLRP3 axis in vivo and in vitro experiments [226]. In summary, a variety of natural products have superior effects in regulating cGAS-STING, inflammasome and pyroptosis pathways, currently being investigated in vitro and in vivo, which should be explored in future research work to provide more diversified options for the treatment of related diseases.

### Synthetic substance

Because of the significance of the STING pathway in the activation of innate immunity and the protection of the host against pathogens, targeting the innate immunity through STING agonists is a potential strategy for both antiviral and antitumor therapies [227, 228]. G10, a human-specific STING agonist, induced STING-dependent activation of both *type I *IFN and the canonical NLRP3 inflammasome in porcine cells [229]. The STING agonist diABZI resulted in cell death and self-DNA release, which was detected by cGAS and formed 2′3’-cGAMP, causing STING hyperactivation, amplifying the TBK1/IRF3 and NF-kB pathways, and subsequent secretion of IFN-I and inflammatory TNFα and IL-6. Meanwhile, the recognition of self-dsDNA or mtDNA by NLRP3 or AIM2 triggered the activation of the inflammasome, thereby leading to the cleavage of the GSDMD, allowing the formation of the GSDMD pore and the release of mature IL-1β and pyroptosis [144]. In traumatic brain injury (TBI), the use of the STING agonist ADU-S100 exacerbated the behavioral and pathological changes [194]. In addition, ADU-S100 promoted microglia activation and exacerbated pyroptosis-associated neuroinflammation by increasing caspase-1 cleavage as well as GSDMD-N-terminal expression [194]. However, administration of the STING antagonist C-176 attenuated TBI-induced inflammatory activation of microglia and reduced pyroptosis [194].

### Recombinant vaccine

A low virulence, ESX-1 effective recombinant BCG vaccine (BCG::ESX-1Mmar) was developed by heterologous expression of the ESX-1 region in BCG, which induced the cGAS-STING-type I IFNs axis and activated the AIM2 and NLRP3 inflammasomes, resulting in a higher proportion of CD8^+^ targeting mycobacterial antigens shared with BCG^+^ T cell effector ratio and specificity of CD4^+^ Th1 cells against ESX-1 antigens [230]. In addition, pyroptosis of DCs via the cGAS-STING pathway and TLRs has recently been shown to be induced by a novel whole-cell inactivated *Pseudomonas aeruginosa* vaccine (XPa) [231]. Artificial nanoparticles, KALA-MENDs, delivered antigen-encoding plasmid DNA (pDNA) to antigen-presenting cells and promoted immune activation, suggesting their use as DNA vaccine vectors [232]. Further studies demonstrated that KALA-MENDs promoted IFN-β and IL-1β secretion through activation of the cGAS-STING pathway and induction of AIM2 and NLRP3 inflammasomes activation [232]. Similarly, a novel virus-like particle was effective at inducing cGAS binding, activating STING signaling, and generating* type I *IFN, and this virus-like particle also induced AIM2 inflammasome formation, GSDMD-mediated pyroptosis, and anti-tumor immunity [233].

### Physical factors

The tumor treating fields (TTFields) is a therapy for the treatment of glioblastoma (GBM) and malignant mesothelioma. In addition, TTFields was found to induce nuclear membrane disruption in microglia, leading to release of large micronuclei from the cells, recruitment and activation of cGAS and AIM2 cytoplasmic DNA sensors, and ultimately leading to activation of the cGAS-STING pathway and the AIM2 inflammasome [234]. TTField-treated GBM cells induced anti-tumor memory immunity and resulted in 42 to 66% cure rates in a STING and AIM2-dependent manner [234]. PtII complexes, Pt1and Pt2, acted as photoactivators of the cGAS-STING pathway, disrupted the mitochondrion and nuclear envelope under light exposure, resulting in cytoplasmic leakage of mtDNA and activation of the cGAS-STING pathway to induce pyroptosis in tumor cells [235]. In addition, activation of the NLRP3 inflammasome and caspase-1 cleavage in macrophages may be promoted by radiation-induced ROS generation or mitochondrial damage [236]. Radiation-induced nuclear DNA leakage into the cytoplasm can be detected by cGAS-STING and activate the immune response; however, knockdown of NLRP3 over-activated the cGAS-STING pathway in macrophages and promoted pyroptosis and radiation-induced tissue damage in mice [237], suggesting that NLRP3 knockdown increases radiation-activated cGAS-STING-mediated IFN-β production, highlighting the importance of fine-tuned regulation.

## Discussion and conclusion

Innate immune responses are rapid responses to disease agents or danger cues that are precisely timed to both effectively combat disease agents and limit excessive inflammation and tissue damage. However, overactivation of innate immunity has been shown to be detrimental and can lead to various diseases. The study of cGAS-STING, inflammasomes and pyroptosis is a rich area within immunology, with rapidly emerging insights into how it works and how to regulate. Due to the similarities in the cGAS-STING, inflammasomes and pyroptosis signaling pathways response to cellular stress and downstream effects, the main review in this paper focuses on their crosstalk network. NLRP3, AIM2 inflammasomes are able to antagonize the cGAS-STING signaling pathway. Upon activation of canonical and non-canonical inflammasomes, caspase-1 could also cleaves cGAS, indicating cross-regulation between intracellular DNA-sensing pathways. Moreover, the cGAS-STING pathway can also be regulated by disrupting the CARD domain of the linker protein ASC in the inflammasome complex. cGAS-STING acts as an important immune axis for microbial infection, chronic inflammation, cancer progression and organ degeneration [1, 9, 18, 238], and also regulates NLRP3, AIM2 inflammasomes.

The cGAS-STING signaling pathway interacts with AIM2 and NLRP3 inflammasome mainly caused by regulatory molecules such as Ox-mtDNA, mtROS, GSDMD, cGAMP and NAT10. mtDNA exposure to ROS induces Ox-mtDNA production, triggering intracytoplasmic NLRP3 inflammasome activation, leading to phosphorylation of STING, which activates the cGAS-STING signaling pathway. AIM2 senses bacterial dsDNA, triggers the formation of the AIM2 inflammasome, leads to the GSDMD cleavage to form membrane pores, thereby limiting bacterial dsDNA binding to cGAS, inhibiting cGAS-STING pathway activation. NAT10 is a negative regulatory factor of neutrophil pyroptosis and overexpression inhibits pyroptosis by blocking the ULK1-STING-NLRP3 pathways. On the other hand, ULK1 has been shown to be involved in NLRP3 autophagy, suggesting that ULK1 has a direct regulatory role on NLRP3 inflammasome in addition to STING inhibition. These key regulatory molecules are critical for the regulation of the crosstalk network of cGAS-STING, inflammasomes, and pyroptosis, and will also provide a strong reference for the selection of therapeutic targets.

Thus, modulating this innate immune system has the potential to treat a broad range of diseases, including infections, neurodegeneration, autoinflammation, metabolic disorders, and cancer. Recent studies have shown that the crosstalk network of cGAS-STING, inflammasomes, and pyroptosis exacerbates cardiac, liver, lung, kidney, spinal cord, nervous system inflammation, induces autoimmune disease and promotes the progression of malignant tumors. While refinement of our understanding of cGAS-STING, inflammasome and pyroptosis continues, targeting of this crosstalk network as a therapeutic for multiple diseases is rapidly progressing. We therefore summarize the involvement of natural products, synthetic substances, recombinant vaccines, and physical factors in regulating the cGAS-STING, inflammasomes and pyroptosis pathways crosstalk network, providing potential candidates for the treatment of related diseases. As the epitome of precision medicine in inflammatory diseases, the continued profiling, refinement and re-purposing of direct and specific modulators will drive future clinical translation.

In summary, the cGAS-STING signaling pathway generates cascade amplification effects between inflammasomes, and pyroptosis, and activates immune inflammatory responses. On the one hand, the crosstalk of these signaling pathways can affect parenchymal organs such as heart, liver, lung, and kidney, and aggravate the development process of inflammatory diseases; in addition, it is also closely related to the progression of several autoimmune diseases. Therefore, further investigations are promising to uncover novel regulatory mechanisms that may provide new opportunities for therapeutic intervention in the exciting field of the crosstalk network of cGAS-STING, inflammasomes and the pyroptosis signaling axis.

## Data Availability

Not applicable.
